# Sulodexide for Secondary Prevention of Recurrent Venous Thromboembolism: A Systematic Review and Meta-Analysis

**DOI:** 10.3389/fphar.2018.00876

**Published:** 2018-08-08

**Authors:** Qing-Jun Jiang, Jun Bai, Jie Jin, Jian Shi, Lefeng Qu

**Affiliations:** ^1^Department of Vascular and Endovascular Surgery, Changzheng Hospital, Second Military Medical University, Shanghai, China; ^2^Department of Gastroenterology, Changzheng Hospital, Second Military Medical University, Shanghai, China

**Keywords:** sulodexide, prevention, recurrence, VTE, extended anticoagulation

## Abstract

**Background:** Patients with venous thromboembolism have high risk of recurrence after discontinuation of anticoagulant treatment. Extended anticoagulation, such as traditional anticoagulants, can reduce the risk of recurrence but is associated with increased risk of hemorrhage. Sulodexide is a natural glycosaminoglycan mixture which can prevent recurrent venous thromboembolism. However, its clinical efficiency and safety still remain controversial.

**Methods:** A systematic search in Medline, EMBASE, Cochrane Library, Web of Science and bibliographies of retrieved articles was performed. Prospective controlled studies reporting the efficacy and safety of sulodexide on the secondary prevention of recurrent venous thromboembolism were included. Two reviewers independently extracted the following data: first author, year of publication, study design, characteristics of patients, data of interventions, doses of sulodexide, overall duration of drug administration, time of follow-up, efficacy and safety outcomes, adverse effects, and the quality of the included studies. The primary efficacy outcomes were recurrent deep vein thrombosis (DVT) or pulmonary embolism. The secondary efficacy outcomes included distal or superficial vein thrombosis and nonfatal or fatal myocardial infarction, stroke, and acute ischemia of the lower limbs. Safety outcome was possible hemorrhagic episodes.

**Results:** Four studies involving 1,461 patients were enrolled in this study. Meta-analysis showed that sulodexide significantly reduced the recurrent venous thromboembolism [RR 0.51, 95 % CI [0.35, 0.74], *P* = 0.0004] and superficial vein thrombosis in the sulodexide group [RR 0.41, 95% CI [0.22, 0.76], *P* = 0.005]. The safety of sulodexide was also reliable. The rate of bleeding was 0.28% in the sulodexide group and 1.60% in the control group, and design of study did not influence these results.

**Conclusions:** Sulodexide could significantly reduce the recurrence of VTE after discontinuation of anticoagulation treatment as compared with placebo.

## Introduction

Venous Thromboembolism (VTE) is a common and potentially fatal condition that includes deep vein thrombosis (DVT) and pulmonary embolism (PE). According to the International Union of Angiology Consensus (Nicolaides et al., 2013), the standard treatment for acute DVT is low-molecular-weight heparin followed by 3–6 months of oral anticoagulation therapy. However, about 25% of patients experience recurrent VTE within 10 years after discontinuation of anticoagulation treatment (Martinez et al., 2014). Of the patients with recurrent proximal DVT, about 30–50% may develop a post-thrombotic syndrome (Prandoni et al., 1997, 2004). Extended treatment with warfarin could reduce the risk of recurrence but increase the risk of bleeding (Kearon et al., 2012; Nordstrom et al., 2015). Clinical trials have shown that the efficacies of new oral anticoagulants (NOACs) and aspirin are non-inferior to warfarin (Becattini et al., 2012; Agnelli et al., 2013; Schulman et al., 2013). However, NOACs maintain to be a bleeding risk and aspirin is not suitable for some patients, such as those with peptic ulcers.

Sulodexide is a natural glycosaminoglycan mixture extracted from porcine intestinal mucosa (Coccheri and Mannello, 2013), and it is safe both for prevention and treatment of thrombotic disorders. It exerts antithrombotic effect by interacting with antithrombin III and heparin cofactor II and by inhibiting thrombin formation. The antithrombotic effect of sulodexide via these two pathways is higher than either component alone. Compared with heparin, sulodexide can be administered orally and it affects the normal hemostasis to a lower extent, leading to low risk of bleeding (Coccheri and Mannello, 2013). Barbanti et al. indicated that sulodexide could prevent venous thrombus formation and promote thrombus dissolution (Barbanti et al., 1992). Thereafter, sulodexide was used for treatment of DVT (Pinto et al., 1997) and prevention of recurrent VTE after discontinuation of anticoagulant therapy (Errichi et al., 2004). Recent studies showed that sulodexide could reduce the risk of recurrent VTE (Luzzi et al., 2014; Andreozzi et al., 2015). However, its clinical efficiency and safety remain controversial.

The aim of this meta-analysis was to evaluate the efficacy and safety of sulodexide in preventing recurrent VTE after effective conventional anticoagulation management.

## Materials and methods

### Search strategy

We searched Medline, EMBASE, Cochrane Library, and Web of Science from inception until Mar. 2018. The following initial search items were used: (“sulodexide”) AND (“thromboembolism” OR “thrombosis” OR “vein” OR “venous”). No language restrictions were imposed. The references from papers were also searched. And the searched results were downloaded for the further screening. The detailed search strategy and presented as a Supplemental Table.

### Inclusion criteria

Included studies must meet the following criteria: (1) study design: prospective controlled studies; (2) population: patients with proximal DVT or PE after anticoagulant treatment. The initial anticoagulant treatment was LMWH (twice daily, weight adjusted), followed by oral anticoagulant for at least 3 months. (3) intervention: the patients in the intervention group were given sulodexide; patients in the control group were given placebo or other anticoagulant agents; and the follow-up period was at least 3 months; (4) the efficacy outcomes were recurrent DVT and PE; and the safety outcome was major or minor bleeding.

### Exclusion criteria

Studies meeting the following items would be excluded: (1) duplicated articles, experimental studies, cohort studies and case-control studies; (2) patients with persistent pulmonary hypertension after PE, those with solid neoplasm or blood disease, those with anti-phospholipid antibody syndrome or antithrombin congenital deficit, patients with New York Heart Association class III to IV cardiorespiratory failure, or patients with known hypersensitivity to glycosaminoglycans.

### Types of outcome measures

The primary efficacy outcomes were recurrent DVT or PE. The secondary efficacy outcomes included distal or superficial vein thrombosis and nonfatal or fatal myocardial infarction, stroke, and acute ischemia of the lower limbs.

The safety outcomes were major or minor bleeding. A major hemorrhage was defined as fatal hemorrhage, a hemorrhage associated with a drop in hemoglobin level of at least 2 g/dL or a need for transfusion of at least 2 units of blood, retroperitoneal hemorrhage, or intracranial hemorrhage. Minor hemorrhage was considered as any other hemorrhagic episode.

### Data extraction

Two reviewers (Qing-Jun Jiang and Jun Bai) independently extracted the following data: first author, year of publication, study design, characteristics of patients, data of interventions, efficacy and safety outcomes, adverse effects, and the quality of included studies. Disagreements were resolved by discussion and consensus. A third reviewer (Jian Shi) was consulted for the decision on inclusion or exclusion for full text evaluation.

### Assessment of methodological quality

The quality of RCT studies was evaluated according to The Cochrane Collaboration's tool for assessing risk of bias: random sequence generation; allocation concealment; blinding of participants, personnel and outcome assessment; incomplete outcome data or selective reporting; and other sources of bias (Deeks et al., 2011). The quality of each item was assessed using the three levels of “low risk” (adequate and correct description of methods or procedures), “high risk” (incorrect description of methods or procedures) or “unclear risk” (no description of methods and procedures).

The Newcastle-Ottawa Scale was used to assess the quality of the non-randomized studies. We made some modifications to the Newcastle-Ottawa Scale to match the needs of this meta-analysis (Zhang et al., 2012). The quality of the studies was evaluated by examining three items: patient selection, comparability of study groups, and assessment of outcome. Studies achieving five or more stars were considered high quality. Methodological quality assessment was independently carried out by two reviewers. Disagreements were resolved by discussion and consensus.

### Statistical analysis

Cochrane Review Manager 5.3 (Cochrane IMS, Oxford, UK) was used for statistical analysis. Outcome measures, such as recurrent VTE, recurrent superficial vein thrombosis, major bleeding, minor bleeding and adverse events, were considered dichotomous variables. Risk ratio (RR), with 95% confidence interval (CI), was used for all primary and secondary dichotomous outcomes by Mantel-Haenszel method. Statistical heterogeneity was assessed by calculating *P*-value and *I*-square (*I*^2^) statistics. Data with *P* < 0.1 and *I*^2^ > 50% as substantial heterogeneity, and a random-effects model was used for the meta-analysis. A fixed-effects model was used for low heterogeneity (*P* ≥ 0.1, *I*^2^ ≤ 50%). Subgroup analyses were performed to explore whether the dosage of sulodexide and the design of study influenced the clinical effect. As the number of trials was small (*n* < 5), funnel plot was not used to assess publication bias.

## Results

### Identification and characteristics of included studies

**Figure 1:**
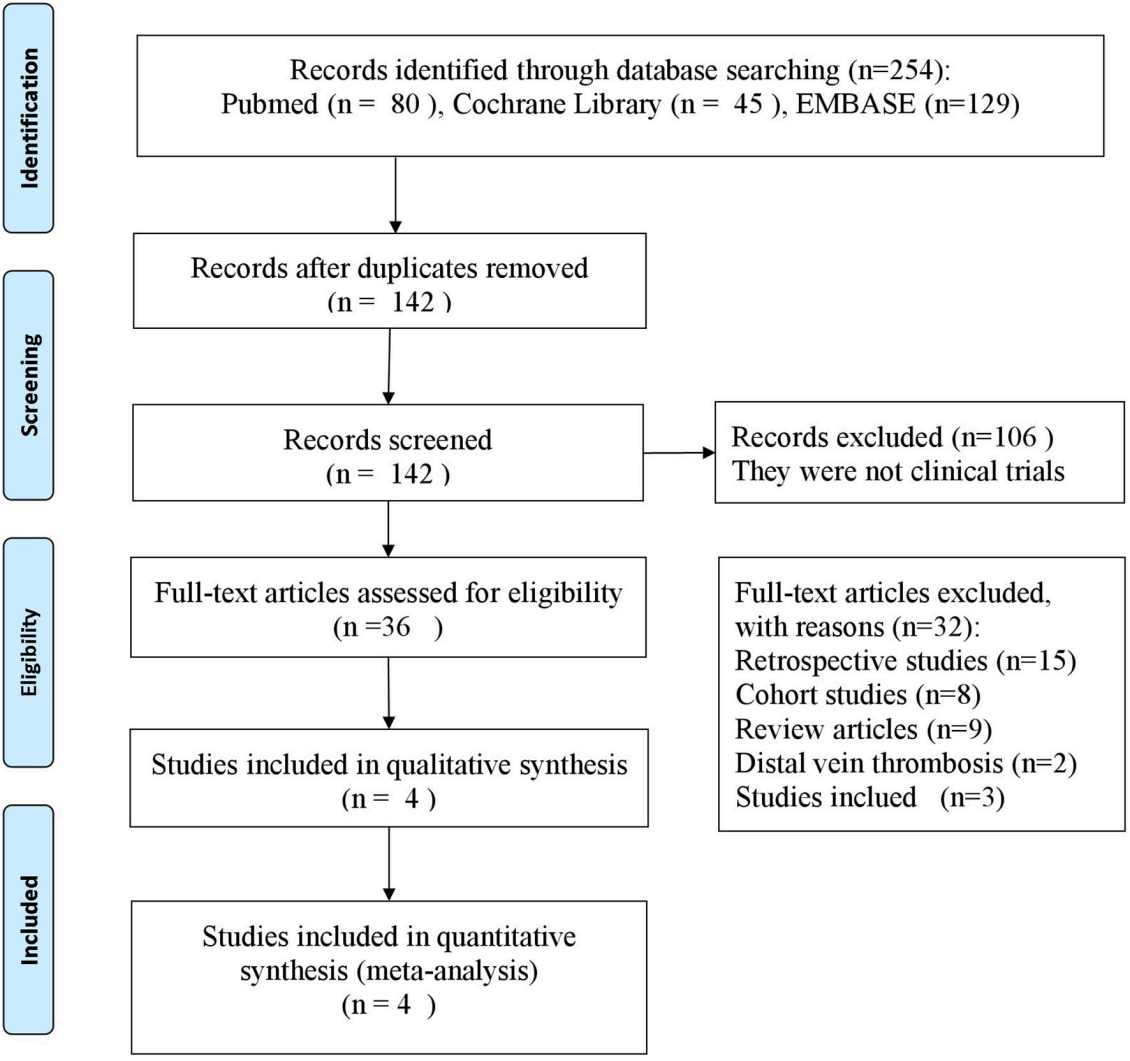
Flow chart of the selection of studies.

We screened the titles and the abstracts of 254 potentially eligible articles (Figure 1). Of these, 112 articles were excluded after duplication review. A total of 106 articles were excluded for not being clinical trials. Twenty-seven articles were excluded for not meeting the objectives of study. We assessed 9 articles in details, of which 5 articles were excluded for the following reasons: different populations (*n* = 2), different anticoagulant agents (*n* = 3). Finally, 4 studies met the inclusion criteria and were included (Errichi et al., 2004; Cirujeda and Granado, 2006; Luzzi et al., 2014; Andreozzi et al., 2015).

The characteristics of these four studies are shown in Table 1. In total, 1,461 patients were involved, of whom 709 were treated with sulodexide and 752 with placebo or other anticoagulant agents. Three studies were prospective randomized controlled studies (Errichi et al., 2004; Cirujeda and Granado, 2006; Andreozzi et al., 2015) and one was prospective non-randomized controlled study (Luzzi et al., 2014). The time of follow-up ranged from 6 to 60 months. Patients received 500 lipasemic units (LSU) of sulodexide twice daily in three studies (Errichi et al., 2004; Luzzi et al., 2014; Andreozzi et al., 2015) and one study used 300 LSU of sulodexide twice daily (Cirujeda and Granado, 2006).

**Table 1:** Characteristics of studies.

**Study author**	**Country**	**Design**	**Subjects**	**Patients(n) S/C**	**Age (m, y) S/C**	**Male *n*, (%)**	**Intervention groups S/C**	**Follow-up**	**Outcomes**
Andreozzi et al., 2015	Italy	RCT	Patients with unprovoked proximal DVT or pulmonary embolism treated with anticoagulant therapy. Age ≥ 18 year	307/308	55.7/55.9	175 (57%)	Sulodexide 500 LSU PO twice daily vs. Placebo	24 months	1. The primary efficacy outcome was recurrent DVT or pulmonary embolism. 2. Secondary efficacy outcomes included distal or superficial vein thrombosis and nonfatal or fatal MI, stroke, or acute ischemia of the lower limbs. 3. Safety outcome was major or clinically relevant nonmajor bleeding.
Cirujeda and Granado, 2006	Spain	RCT	Patients with proximal DVT of the lower limbs treated with anticoagulant therapy. Age ≥ 18 year	75/75	67.7/66.1	92 (61.3%)	Sulodexide 300 LSU PO twice daily vs. Acenocoumarol 2 mg/day	6 months	1. The primary efficacy outcome was recurrent DVT or pulmonary embolism. 2. Safety outcome was possible hemorrhagic episodes
Errichi et al., 2004	Italy	RCT	Patients with DVT treated with anticoagulant therapy.	202/203	53.2/52.2	NR	Sulodexide 500 LSU PO twice daily vs. Placebo	24 months	1. The primary efficacy outcome was recurrent DVT or pulmonary embolism. 2. Secondary efficacy outcomes included distal or superficial vein thrombosis and nonfatal or fatal MI, stroke, or acute ischemia of the lower limbs. 3. Safety outcome was major or clinically relevant nonmajor bleeding.
Luzzi et al., 2014	Italy	PC	Patients with proximal DVT	124/167	47.2/46.4	131 (45.0%)	Sulodexide 500 LSU PO twice daily vs. Placebo	60 months	1.The primary efficacy outcome was recurrent DVT or pulmonary embolism. 2.Safety outcome was possible hemorrhagic episodes

*S, sulodexide group; C, control group; PO, per os; NR, not reported*.

*DVT, deep vein thrombosis; LSU, lipoprotein lipase releasing units*.

*RCT, prospective randomized controlled trials; PC, prospective non-randomized controlled study*.

### Quality assessment of included studies

**Figure 2:**
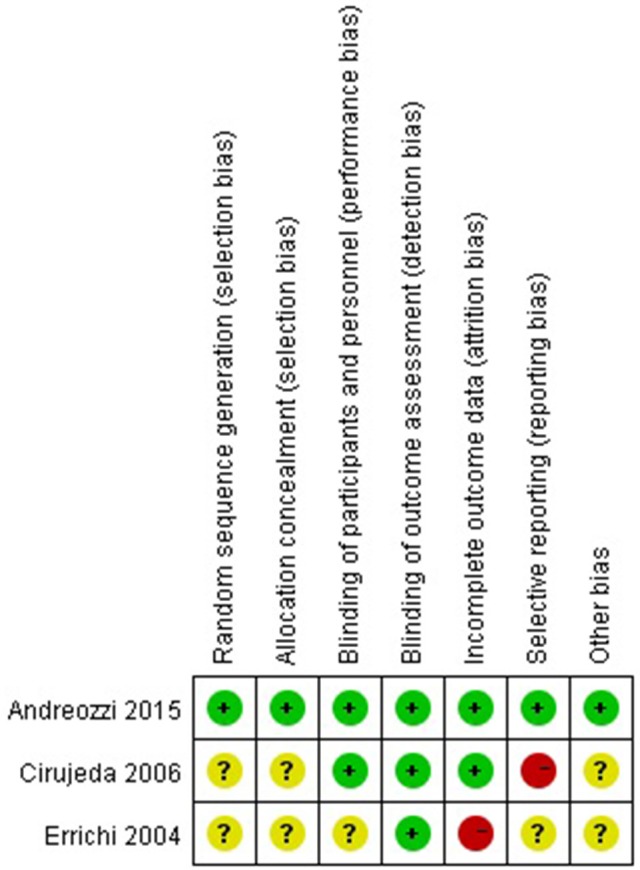
Methodological quality assessment of the risk of bias for each included study. Green circles indicate low risk of bias, yellow circles unclear risk of bias, and red circles high risk of bias.

The quality assessment of RCT studies is summarized in Figure 2. All the three RCT studies mentioned randomized allocation. One mentioned the appropriate generation of the random allocation sequence and concealment (Andreozzi et al., 2015). All the three RCT studies stated blinding of outcome assessment (Errichi et al., 2004; Cirujeda and Granado, 2006; Andreozzi et al., 2015). One study had high risk of incomplete outcome (Errichi et al., 2004). One study had high risk of selective reporting bias (Cirujeda and Granado, 2006).

The Newcastle-Ottawa Scale was used to judge non-randomized controlled study. The non-randomized controlled study scoring seven stars was considered high quality (Luzzi et al., 2014) (Table 2).

**Table 2:** Check list for methodological quality assessment.

**Selection**	**Study score**
1. Assignment for treatment: any criteria reported? (Yes one star)	^*^
2. How representative was the sulodexide group in comparison with the population with venous thromboembolism? (Yes one star)	^*^
3. How representative was the control group? (Yes one star)	^*^
**Comparability**	
4. Study controls for prevention of recurrent venous thromboembolism? (Yes one star)	^*^
5. Study controls for the first episode of unprovoked venous thromboembolism? (No)	^*^
**Outcome Assessment**	
6. Clearly reported following outcomes: recurrent DVT postprocedural complications? (Yes one star)	^*^
7. Assessment of outcome: blind record linkage? (No)	
8. Were the outcomes analyzed according to the intention to-treat principle? (Yes one star)	^*^
9. Adequacy of follow-up > 90%? (Yes, one star)	^*^

*The star means the score of included study*.

## Meta-analysis

### Prevention of primary efficacy outcomes with sulodexide

#### The overall recurrent VTE

Firstly, we performed an overall analysis based on primary efficacy outcome data from the last follow-up. Regardless of the study design and the dose of sulodexide, recurrent VTE was noticed in 37 of the 709 participants (5.22%) in the sulodexide group compared with 76 of the 752 participants (10.11%) in the control group. Meta-analysis showed that sulodexide significantly reduced the recurrence of VTE [RR 0.51, 95% CI [0.35, 0.74], *P* = 0.0004].

When the primary efficacy outcomes were separated as recurrent DVT and PE events, recurrent DVT occurred in 34 of the 671 participants (5.07%) in the sulodexide group compared with 72 of the 711 participants (10.13%) in the control group. Meta-analysis showed that sulodexide significantly reduced the recurrence of DVT [RR 0.49, 95% CI [0.33, 0.73], *P* = 0.0004].

**Figure 3:**
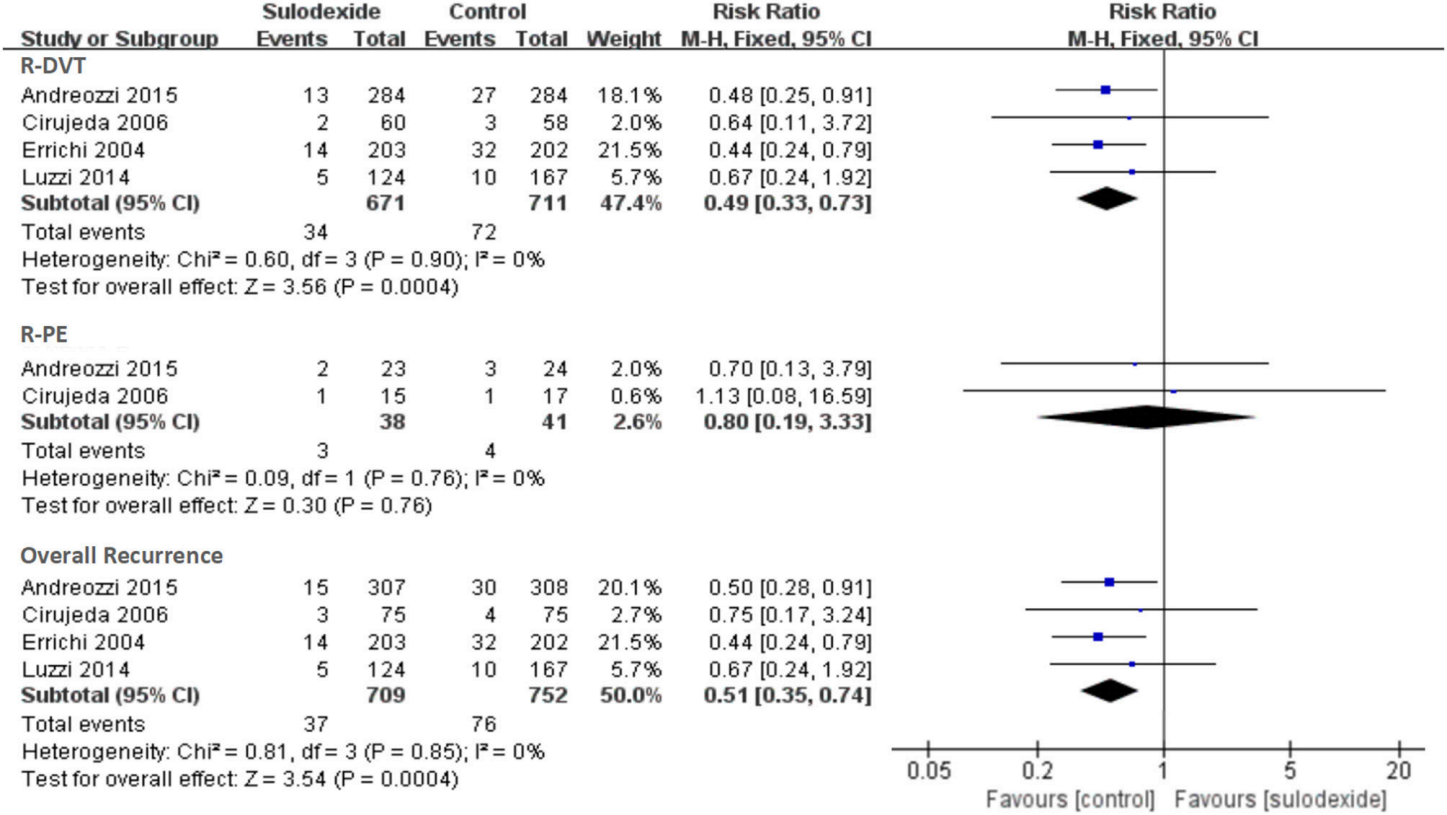
Recurrence of deep vein thrombosis and pulmonary embolism. Forest plot includes pooled estimates for studies comparing sulodexide to control for prevention of recurrent venous thromboembolism.

Two studies reported the recurrent PE (Cirujeda and Granado, 2006; Andreozzi et al., 2015). Recurrent PE was found in 3 of the 38 participants (7.89%) in the sulodexide group compared with 4 of the 41 participants (9.76%) in the control group. Meta-analysis showed that there was no difference in the recurrent PE events between the two groups [RR 0.80, 95 % CI [0.19, 3.33], *P* = 0.76] (Figure 3).

#### Prevention of recurrent VTE at 6-mon follow-up

**Figure 4:**
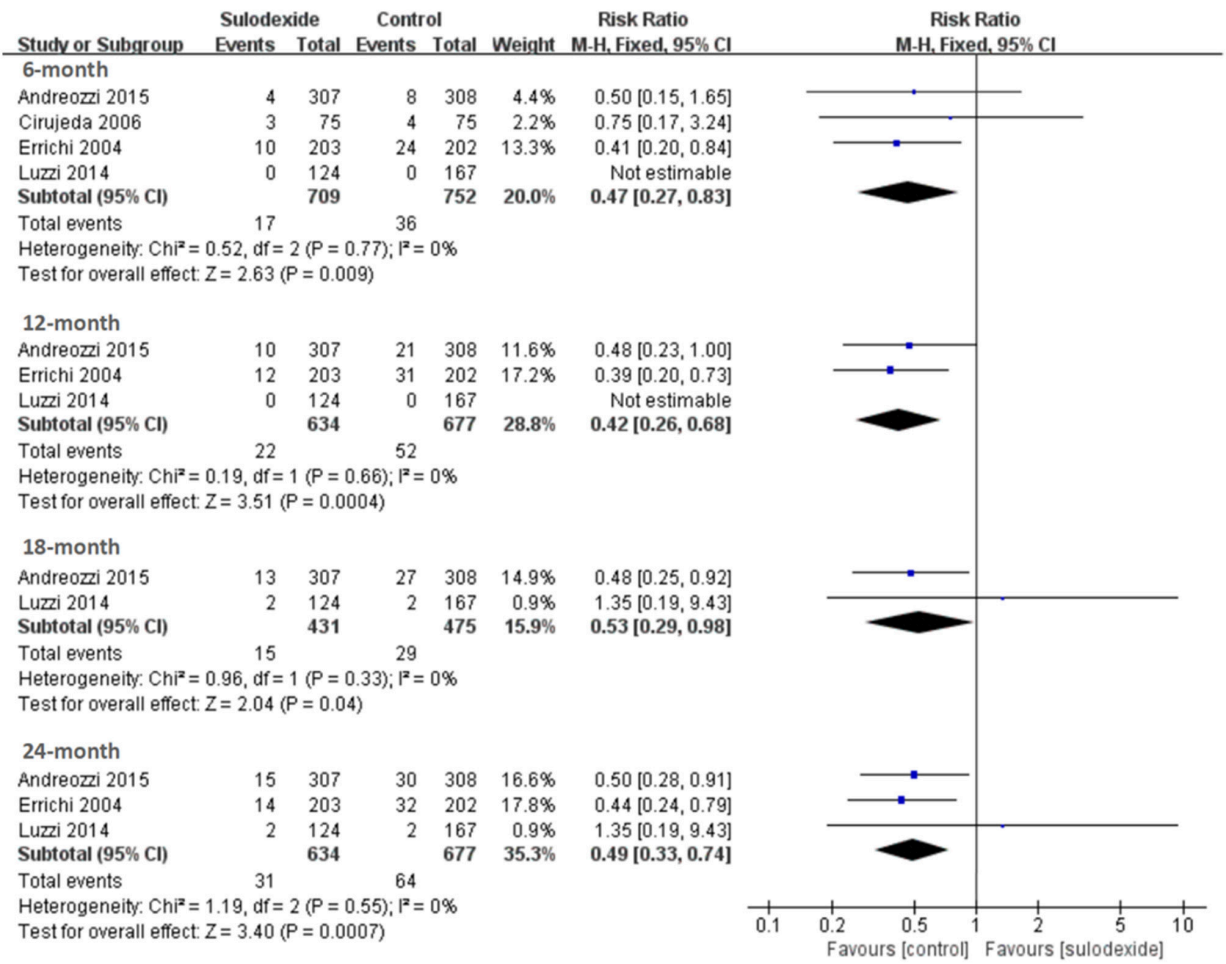
Recurrent venous thromboembolism at different follow-up periods.

All studies (Errichi et al., 2004; Cirujeda and Granado, 2006; Luzzi et al., 2014; Andreozzi et al., 2015) reported the 6 months' follow-up outcomes. The recurrence rates of VTE in the sulodexide group and the control group were 2.40 and 4.79%, respectively. Pooling of these studies showed low recurrence of DVT in participants treated with sulodexide [RR 0.47, 95% CI [0.27, 0.83], *P* = 0.009] (Figure 4).

#### Prevention of recurrent VTE at 12-mon follow-up

Three studies (Errichi et al., 2004; Luzzi et al., 2014; Andreozzi et al., 2015) reported 12 months' follow-up outcomes. The recurrence rates of VTE in the experimental group and the control group were 3.47 and 7.68%, respectively. Pooling of these studies showed low recurrence of DVT in patients treated with sulodexide [RR 0.42, 95 % CI [0.26, 0.68], *P* = 0.0004] (Figure 4, Table 3).

**Table 3:** Recurrent VTE at different follow-up periods.

	**No. studies**	**No. patients**	**Recurrence rate (%) (sulodexide/control)**	**RR (95% CI)**	***P*****-value**
6-month follow-up	4	1,461	2.40/4.79	0.47 [0.27, 0.83]	0.009
12-month follow-up	3	1,311	3.47/7.68	0.42 [0.26, 0.68]	0.0004
18-month follow-up	2	906	3.48/6.11	0.53 [0.29, 0.98]	0.04
24-month follow-up	3	1,311	4.89/9.45	0.49 [0.33, 0.74]	0.0007

*RR, relative risk; CI, confidence intervals*.

#### Prevention of recurrent VTE at 18-mon follow-up

Two studies (Luzzi et al., 2014; Andreozzi et al., 2015) reported 18 months' follow-up outcomes. The recurrence rates of VTE in the experimental group and the control group were 3.48 and 6.11%, respectively. Pooling of these studies showed low recurrence of DVT in patients treated with sulodexide [RR 0.53, 95% CI [0.29, 0.98], *P* = 0.04] (Figure 4, Table 3).

#### Prevention of recurrent VTE at 24-mon follow-up

Three studies (Errichi et al., 2004; Luzzi et al., 2014; Andreozzi et al., 2015) reported 24 months' follow-up outcomes. The recurrence rates of VTE in the experimental group and the control group were 4.89 and 9.45%, respectively. Pooling of these studies showed low recurrence of DVT in patients treated with sulodexide [RR 0.49, 95 % CI (0.33, 0.74), *P* = 0.0007] (Figure 4, Table 3).

### Prevention of secondary efficacy outcomes with sulodexide

**Figure 5:**
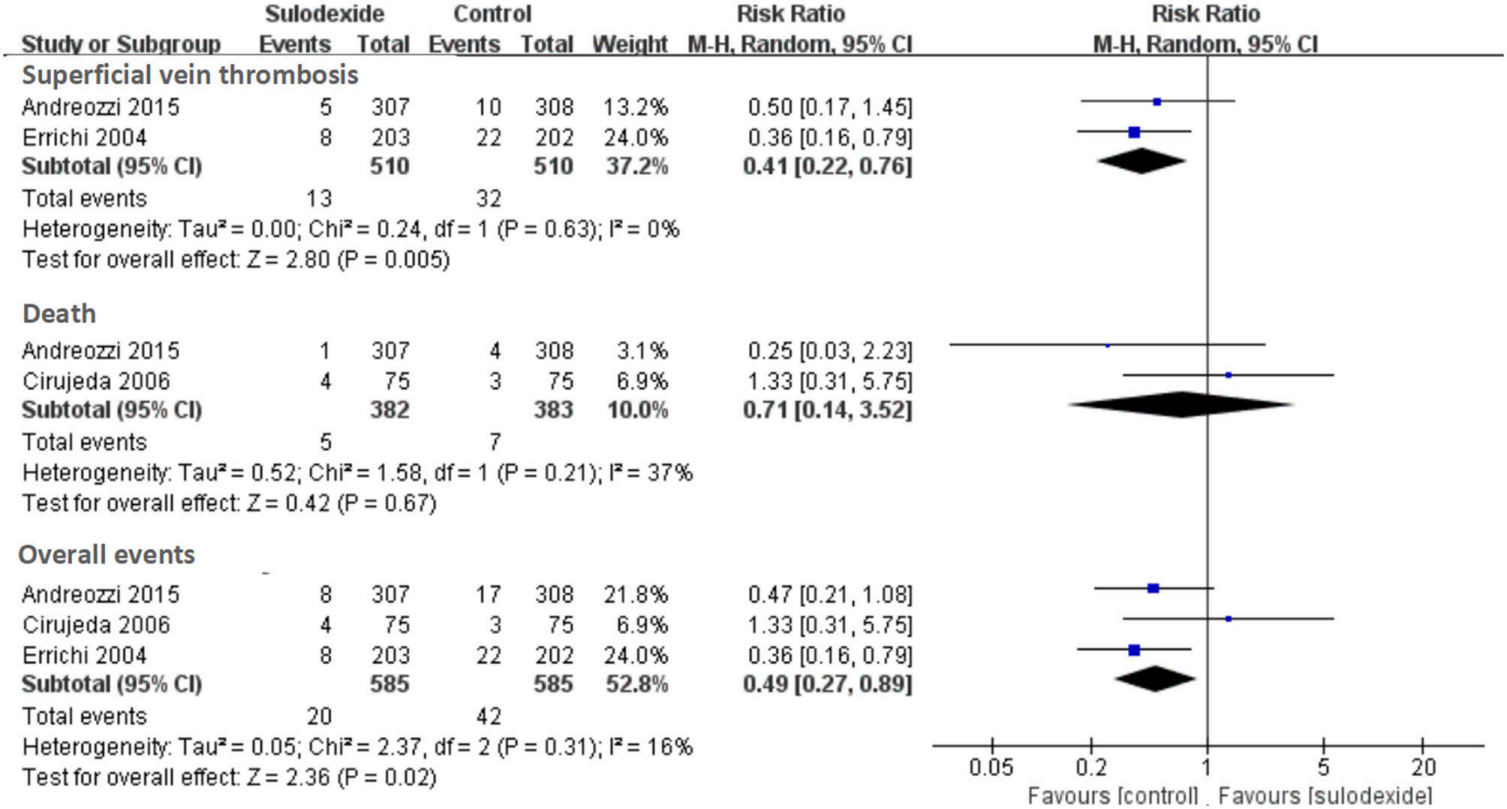
Meta-analysis of secondary efficacy outcomes.

Secondary efficacy outcomes included distal or superficial vein thrombosis and nonfatal or fatal myocardial infarction, stroke, or acute ischemia of the lower limbs. A total of three studies reported sulodexide in the prevention of secondary efficacy outcomes (Errichi et al., 2004; Cirujeda and Granado, 2006; Andreozzi et al., 2015). Meta-analysis of these three studies indicated significant reduction of secondary efficacy outcomes in the sulodexide group [RR 0.49, 95% CI [0.27, 0.89], *P* = 0.02] (Figure 5).

Two studies reported sulodexide for the prevention of superficial vein thrombosis (Errichi et al., 2004; Andreozzi et al., 2015). Meta-analysis indicated significant reduction of superficial vein thrombosis in the sulodexide group [RR 0.41, 95% CI [0.22, 0.76], *P* = 0.005] (Figure 5). Two studies reported the fatal myocardial infarction and death (Cirujeda and Granado, 2006; Andreozzi et al., 2015). There was no difference in severe adverse events between the two groups (RR 0.71, 95% CI [0.14, 3.52], *P* = 0.67] (Figure 5).

### Risk of hemorrhagic episodes after exposure to sulodexide

All studies reported the safety outcomes. There were only 2 (0.28%) cases of minor bleeding in the sulodexide group (Andreozzi et al., 2015), compared with 1 (0.13%) case of major bleeding and 11 (1.46%) cases of minor bleeding in the control group (Cirujeda and Granado, 2006; Andreozzi et al., 2015) (Table 4).

**Table 4:** Hemorrhagic complications.

	**Sulodexide**		**Control**	
	**Major bleeding**	**Minor bleeding**	**Major bleeding**	**Minor bleeding**
Andreozzi et al., 2015	2	0	0	2
Cirujeda and Granado, 2006^*^	0	0	1	9
Errichi et al., 2004	0	0	0	0
Luzzi et al., 2014	0	0	0	0
Total	2		12	

* *The control drug was acenocoumarol*.

### Adverse events

The adverse events of these three studies are summarized in in Table 5 (Errichi et al., 2004; Cirujeda and Granado, 2006; Andreozzi et al., 2015). In sulodexide group, a total of 11 participants developed adverse events, of which 6 had upper abdominal pain and 5 had anaphylaxis characterized by pruritus. In the control group, adverse reactions were found in 18 cases, including epigastric pain (5), nausea (4), vomiting (5), and allergic reactions (4).

**Table 5:** Adverse events.

	**Sulodexide**				**Control**			
	**Abdominal pain upper**	**Nausea**	**Vomiting**	**Allergy**	**Abdominal pain upper**	**Nausea**	**Vomiting**	**Allergy**
Andreozzi et al., 2015	6	0	0	4	5	4	5	1
Cirujeda and Granado, 2006^*^	0	0	0	1	0	0	0	3
Errichi et al., 2004	0	0	0	0	0	0	0	0
Total	11				18			

* *The control drug was acenocoumarol*.

## Subgroup analysis

### Doses of sulodexide

**Figure 6:**
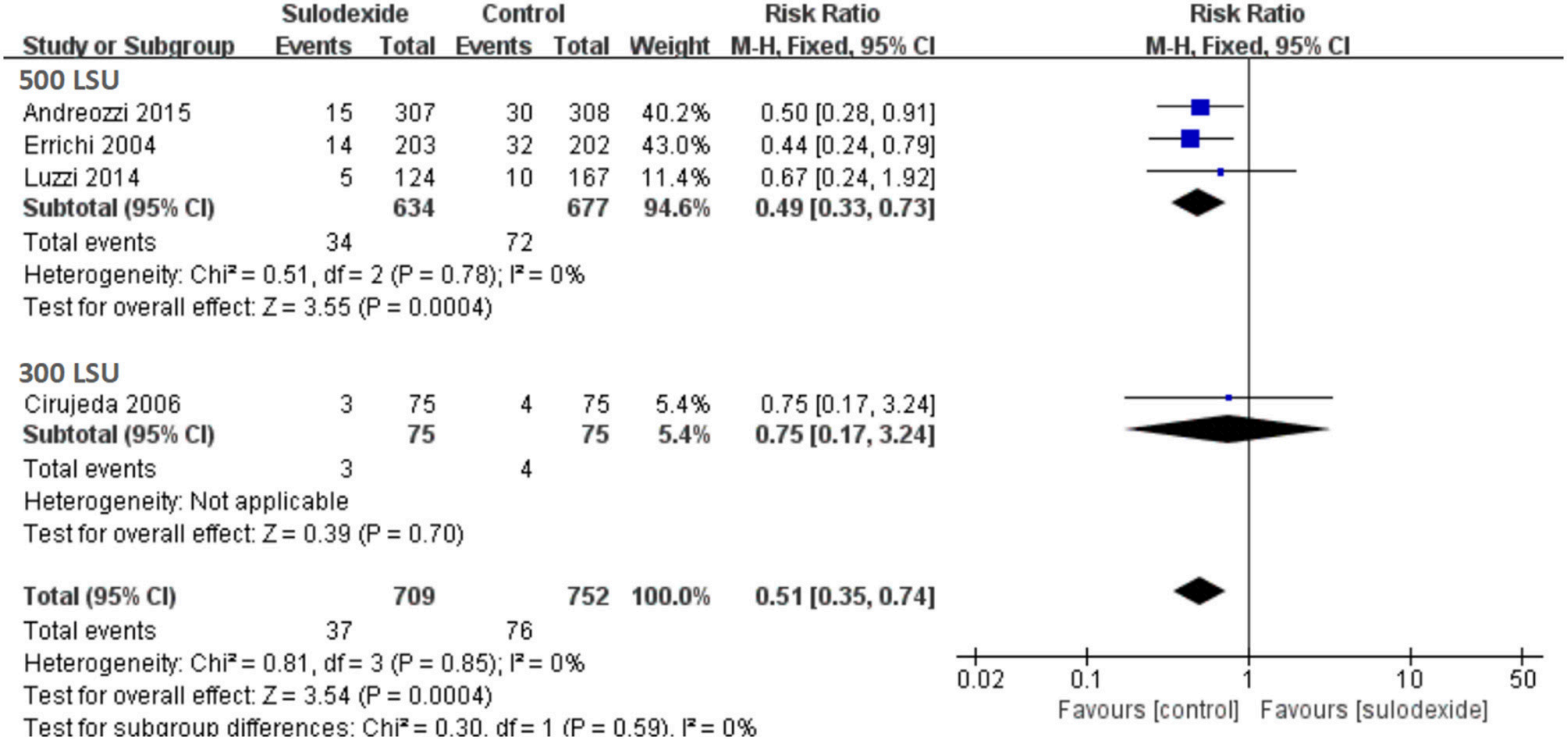
Different doses of sulodexide for prevention of recurrent venous thromboembolism.

Since different doses of sulodexide were used in the four studies, we therefore performed subgroup analyses. A total of three studies used the oral sulodexide with 500 LSU and one study used the oral sulodexide with 300 LSU. When patients received 500 LSU sulodexide (Errichi et al., 2004; Luzzi et al., 2014; Andreozzi et al., 2015), the recurrence rate of the sulodexide group (5.36%) was significantly lower than that of the control group (10.64%) [RR 0.49, 95 % CI [0.33, 0.73], *P* = 0.0004]. Comparing the sulodexide 300 LSU with acenocoumarol, we found no difference between the two groups [RR 0.75, 95% CI [0.17, 3.24], *P* = 0.70] (Figure 6).

### Study design

**Figure 7:**
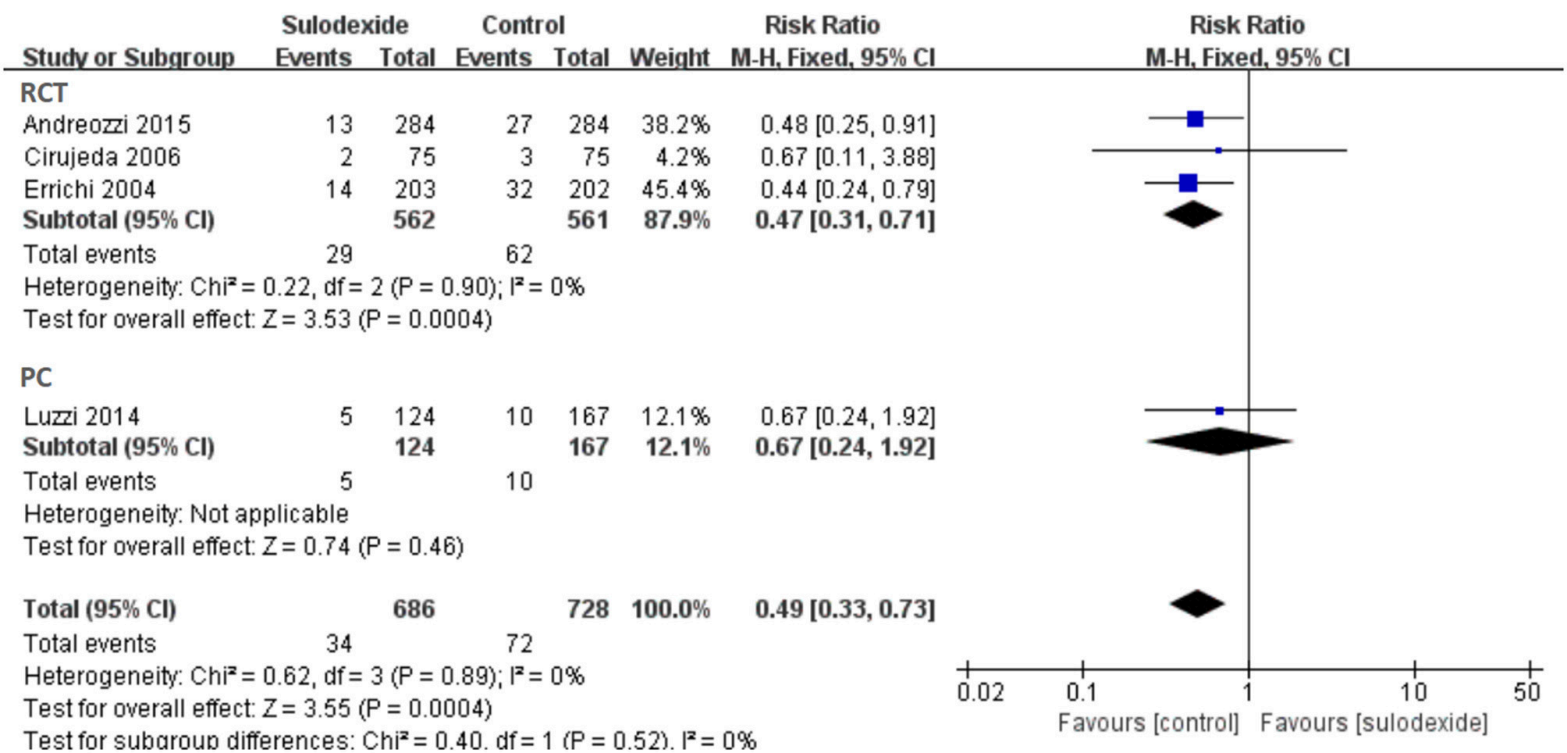
Different study designs of sulodexide for prevention of recurrent deep venous thrombosis.

Due to different study designs in the four studies, we therefore performed subgroup analyses. A total of three randomized clinical trials and one prospective non-randomized comparative study were included. When we excluded the non-randomized comparative study, the recurrent rate of DVT of the sulodexide group was significantly lower than that of the control group [RR 0.47, 95% CI [0.31, 0.71], *P* = 0.0004]. In the prospective non-randomized comparative study, there was no difference between the two groups [RR 0.67, 95% CI [0.24, 1.92], *P* = 0.46] (Figure 7).

## Discussion

Even after effective conventional anticoagulation management, the recurrence rate of VTE is relatively high. Therefore, extended anticoagulation should be considered to prevent recurrent VTE. We evaluated the effects of sulodexide for the secondary prevention of recurrent VTE. Our results indicated that sulodexide significantly reduced the recurrence of VTE (5.22 vs. 10.11%), without increasing the bleeding risk. The safety of sulodexide is also reliable. The rate of bleeding was 0.28% in the sulodexide group and 1.60% in the control group. Furthermore, our meta-analysis did not find any significant difference in adverse complications between the two groups.

Sulodexide is a natural glycosaminoglycan mixture consisting of 80% fast-moving heparin and 20% dermatan sulfate. Sulodexide exerts its antithrombotic effect by interacting with antithrombin III and heparin cofactor II and by the inhibiting thrombin formation (Coccheri and Mannello, 2013; Hoppensteadt and Fareed, 2014). Sulodexide also exerts fibrinolytic activity by promoting the release of tissue plasminogen activator (tPA) and reducing the activity of plasminogen activator inhibitor (Ofosu, 1998), and thereby does not change the activated partial thromboplastin time (Mauro et al., 1993). Using a rat venous thrombosis model Barbanti et al. found that sulodexide could not only prevent venous thrombus formation, but also promote thrombus dissolution (Barbanti et al., 1992). In 1993, sulodexide was used as an antithrombotic agent for chronic venous diseases (Saviano et al., 1993). Thereafter, sulodexide began to be used in the treatment of DVT (Pinto et al., 1997) and prevention of recurrent VTE after discontinuation of anticoagulant therapy (Errichi et al., 2004). These studies have demonstrated the effectiveness of sulodexide in the treatment of chronic venous diseases.

Since the follow-up period of the included studies ranged from 6 months to 60 months, we then performed analyses of the different follow-up periods. Not surprisingly, the results confirmed that the recurrence rate of VTE increased year by year. At two-year follow-up, the recurrence rate of VTE in the sulodexide group and the control group were up to 4.89 and 9.45%, respectively. Pooling of these studies showed low recurrence of DVT in patients treated with sulodexide. A previous study showed that sulodexide was effective in the treatment of superficial thrombophlebitis (Messa et al., 1997). Our meta-analysis revealed that sulodexide could significantly reduce the incidence of superficial vein thrombosis, thereby improving the Health-Related Quality of Life (HRQOL) of patients.

The recommended dose of sulodexide was 500 LSU and the sensitivity analyses also proved that oral 500 LSU sulodexide could significantly reduce the recurrence of VTE. However, it is important to point out that the recurrence rate of VTE for the dose 300 LSU is also low (4.0%) (Cirujeda and Granado, 2006), suggesting that 300 LSU of sulodexide might be enough in secondary prevention of recurrent VTE. However, a large-size RCT is needed to evaluate the efficacy of 300 LSU sulodexide for prevention of recurrent VTE.

These findings showed that sulodexide was valuable in preventing recurrent VTE after discontinuation of anticoagulant treatment. To our knowledge, warfarin and NOACs are widely used in secondary prevention of recurrent VTE. If our results are correct, patients with VTE will benefit from extended treatment with sulodexide, and physicians will have an auxiliary method to prevent the recurrent VTE, without worrying about increased bleeding risk. However, randomized trials are still needed to further evaluate the efficacy and safety of sulodexide compared with NOACs or aspirin in secondary prevention of recurrent VTE.

Prior meta-analyses examined the effects of warfarin, aspirin and some NOACs on the recurrent VTE. Our study was the first meta-analysis focusing on the role of sulodexide in secondary prevention of recurrent VTE. We analyzed the recurrent VTE at different follow-up periods. Furthermore, we also performed subgroup analyses to explore whether the dosage of sulodexide and the design of study influenced the clinical effects. Despite these findings, our meta-analysis had several limitations. Firstly, although there was no heterogeneity between studies, the small number of included trials would have reduced the statistical power of the analysis. In addition, all the four included studies were of high quality, but one was non-randomized study. Furthermore, some studies did not report important outcomes, such as adverse events. Finally, the data of this meta-analysis were extracted and summarized from each study publications, so it might contain selection and publication bias.

## Conclusion

In this study, it has been shown that sulodexide can significantly reduce the rate of recurrent DVT as compared with placebo.

## Author contributions

Q-JJ, JB, and JJ contributed equally to this work. Q-JJ and JB analyzed the data and wrote the manuscript. Q-JJ and JJ collected the data and performed the analyses. JS and LQ designed the study and amended the paper.

### Conflict of interest statement

The authors declare that the research was conducted in the absence of any commercial or financial relationships that could be construed as a potential conflict of interest. The reviewer ED and handling Editor declared their shared affiliation.
